# Prevalence of dementia among elderly Brazilians: a systematic review

**DOI:** 10.1590/S1516-31802011000100009

**Published:** 2011-01-06

**Authors:** Susana Dytz Fagundes, Marcus Tolentino Silva, Maria Fernanda Reis Silva Thees, Maurício Gomes Pereira

**Affiliations:** I MD, MSc. Gynecologist and Obstetrician, Universidade Católica de Brasília, Brasília, Federal District, Brazil.; II Pharmacist. Adviser, Department of Science and Technology, Secretariat of Science, Technology and Strategic Inputs, Ministry of Health, Brasília, Federal District, Brazil.; III Biologist. Adviser, National Health Surveillance Agency (Anvisa), Ministry of Health, Brasília, Brazil.; IV MD, PhD. Full Professor, School of Medicine, Universidade de Brasília, Federal District, Brazil.

**Keywords:** Aged, Memory disorders, Frail elderly, Memory, Dementia, Prevalence, Idoso, Transtornos da memória, Idoso fragilizado, Memória, Demência, Prevalência

## Abstract

**CONTEXT AND OBJECTIVE:**

The rapid growth of the elderly population in Brazil has implied a significant rise in the number of individuals with dementia. The real prevalence of this disease needs to be ascertained in order to establish appropriate measures for improving the quality of life of those affected. The aim of this study was to estimate the prevalence of dementia among elderly Brazilians (≥ 60 years) through a systematic review of high-quality, population-based, double-blind studies published between 1990 and 2010.

**DESIGN AND SETTING:**

Systematic review of prevalence studies. The manuscript was produced in the School of Medicine, Universidade de Brasília.

**METHODS:**

Database searches for articles were conducted in Medline (Medical Literature Analysis and Retrieval System Online), Embase (Excerpta Medica database), Lilacs (Literatura Latino-Americana e do Caribe em Ciências da Saúde), SciELO (Scientific Electronic Library Online) and theses and dissertations, using specific keywords. Quality was assessed according to eight criteria for sampling and measurement of findings.

**RESULTS:**

Out of 112 articles identified, eleven were included in the qualitative synthesis. In five higher-quality studies from São Paulo, the prevalence rates ranged from 5.1 to 19.0%. No meta-analysis was presented because of heterogeneity among the studies. Poor, illiterate, female and very elderly individuals were the groups most affected.

**CONCLUSION:**

The findings from this review did not reflect the reality of the whole country. Although the results brought some information on the prevalence and distribution of dementia in Brazil, cross-sectional studies with consistent methodology are needed.

## RESUMO

**CONTEXTO E OBJETIVO:**

O crescimento rápido da população idosa no Brasil implicou no aumento significativo do número de indivíduos com demência. A prevalência real da doença deve ser constatada a fim de que se possam estabelecer medidas apropriadas para melhorar a qualidade de vida das pessoas afetadas. O objetivo deste estudo foi estimar a prevalência de demência entre a população brasileira idosa (≥ 60 anos) através da revisão sistemática de estudos populacionais, duplo-cegos, de alta qualidade, publicados entre 1990 e 2010.

**TIPO DE ESTUDO E LOCAL:**

Revisão sistemática de estudos de prevalência. O manuscrito foi realizado na Faculdade de Medicina, Universidade de Brasília.

**MÉTODOS:**

Os artigos foram pesquisados nos bancos de dados Medline (Medical Literature Analysis and Retrieval System Online), Embase (Excerpta Medica database), Lilacs (Literatura Latino-Americana e do Caribe em Ciências da Saúde), SciELO (Scientific Electronic Library Online) e de teses e dissertações, usando-se palavras-chave específicas. A qualidade foi avaliada de acordo com oito critérios relacionados à amostragem e à mensuração dos achados.

**RESULTADOS:**

Dentre os 112 artigos identificados, 11 foram incluídos na síntese qualitativa. Em cinco estudos de melhor qualidade realizados em São Paulo, a taxa de prevalência variou de 5,1% a 19,0%. A metanálise não foi realizada devido à heterogeneidade entre os estudos. Pobres, analfabetos, mulheres e pessoas de idade avançada foram os grupos mais afetados.

**CONCLUSÃO:**

Os achados desta revisão não refletem a realidade de todo o país. Embora os resultados mostrem informações sobre a prevalência e a distribuição da demência no Brasil, estudos transversais com metodologia consistente precisam ser realizados.

## INTRODUCTION

The average age of the Brazilian population is increasing rapidly, and the fastest growing sector consists of adults ≥ 80 years of age.^1-3^ According to data derived from the demographic census, there were just two million elderly individuals (≥ 60 years old) in Brazil in 1950, comprising 4% of the country's total population. By 2000, this figure had risen to 14.5 million. i.e. 8.6% of the total population. It is estimated that the elderly will account for 25% of the Brazilian population by 2050. These demographic dynamics will give rise to changes in the health profile of the population, consisting especially of increases in the prevalence of chronic degenerative diseases and physical and mental incapacities. An aging population implies intensification of the demand for social and health services. This poses a significant challenge for public policies.

Dementia is one of the most distressing health problems that affect the elderly. It destroys individuals' and families' quality of life.^4^ Although the problem of dementia is a matter of concern for government authorities, data relating to the prevalence of the disease in Brazil are scarce. The objective of this systematic review was to draw up a profile of the prevalence of dementia in this country.

## METHODS

### Electronic search and selection of articles for review

Relevant articles published during the period 1990-2010 were retrieved from traditional electronic databases, including Medline (Medical Literature Analysis and Retrieval System Online) (via PubMed), Embase (Excerpta Medica database), Lilacs (Literatura Latino-Americana e do Caribe em Ciências da Saúde) (via Biblioteca Virtual de Saúde, BVS), SciELO (Scientific Electronic Library Online) and the Brazilian thesis database. The keywords were in Portuguese, Spanish and English and related to dementia, elderly, prevalence and Brazil (**Table 1**). Population-based cross-sectional studies with the use of cognitive tests to evaluate dementia among individuals > 60 years old were included in the review. Studies were excluded if they dealt with subjects with psychiatric disorders, cognitive impairment due to congenital, neurological or infectious causes, long-term dementia or test validation. The initial selection of the articles was based on analysis of the abstracts, and was performed independently by three researchers (SDF, MTS, MFRST). The references of the articles selected were evaluated to identify further published papers. Decisions concerning the inclusion or exclusion of articles were made jointly by all the researchers.

**Table 1.** Full electronic search strategy

Database	Search terms
Medline	(“Aged”[Mesh] OR Elderly OR “Aged, 80 and over”[Mesh] OR (Oldest Old) OR Nonagenarians OR Nonagenarian OR Octogenarians OR Octogenarian OR Centenarians OR Centenarian) AND (“Dementia”[Mesh] OR Dementias OR Amentia OR Amentias OR (Senile Paranoid Dementia) OR (Dementias, Senile Paranoid) OR (Paranoid Dementia, Senile) OR (Paranoid Dementias, Senile) OR (Senile Paranoid Dementias) OR (Familial Dementia) OR (Dementia, Familial) OR (Dementias, Familial) OR (Familial Dementias)) AND (“Prevalence”[Mesh] OR Prevalences) AND (/Brazil)
Embase	#1 ‘aged’/exp AND [embase]/lim #2 ‘dementia’/exp AND [embase]/lim #3 ‘prevalence’/exp AND [embase]/lim #4 ‘brazil’/exp AND [embase]/lim #5 #1 AND #2 AND #3 AND #4
Lilacs	(Aged OR Elderly OR Anciano OR (Adulto Mayor) OR Idoso OR (Aged, 80 and over) OR Centenarians OR Nonagenarians OR Octogenarians OR (Oldest Old) OR (Anciano de 80 o más Años) OR Centenarios OR Nonagenarios OR Octogenarios OR Viejísimos OR (Anciano de 80 Años o más) OR (Ancianos de 80 o más Años) OR (Ancianos de 80 Años o más) OR (Ancianos de 80 Años y más) OR (Idoso de 80 Anos ou mais) OR Centenários OR Nonagenários OR Octogenários OR Velhíssimos OR (Idoso de 80 ou mais Anos) OR (Idosos de 80 ou mais Anos) OR (Idosos de 80 Anos ou mais)) AND (Dementia OR (Senile Paranoid Dementia) OR Demencia OR (Demencia Paranoide Senil) OR Demência OR (Demência Senil Tipo Paranóide)) AND (Prevalence OR (Prevalence Rate) OR Prevalencia OR (Tasa de Prevalencia) OR (Coeficiente de Prevalencia) OR Prevalência OR (Taxa de Prevalência) OR (Coeficiente de Prevalência)) AND (Brazil OR Brasil)
SciELO	(Aged OR Elderly OR Anciano OR (Adulto Mayor) OR Idoso OR (Aged, 80 and over) OR Centenarians OR Nonagenarians OR Octogenarians OR (Oldest Old) OR (Anciano de 80 o más Años) OR Centenarios OR Nonagenarios OR Octogenarios OR Viejísimos OR (Anciano de 80 Años o más) OR (Ancianos de 80 o más Años) OR (Ancianos de 80 Años o más) OR (Ancianos de 80 Años y más) OR (Idoso de 80 Anos ou mais) OR Centenários OR Nonagenários OR Octogenários OR Velhíssimos OR (Idoso de 80 ou mais Anos) OR (Idosos de 80 ou mais Anos) OR (Idosos de 80 Anos ou mais)) AND (Dementia OR (Senile Paranoid Dementia) OR Demencia OR (Demencia Paranoide Senil) OR Demência OR (Demência Senil Tipo Paranóide)) AND (Prevalence OR (Prevalence Rate) OR Prevalencia OR (Tasa de Prevalencia) OR (Coeficiente de Prevalencia) OR Prevalência OR (Taxa de Prevalência) OR (Coeficiente de Prevalência)) AND (Brazil OR Brasil)
Brazilian thesis database*	Demência Filters: “Tipo de mídia: texto”; “Categoria: teses e dissertações”

* available at www.dominiopublico.gov.br (only in Portuguese)

### Appraisal of the articles, data extraction and analysis

The articles selected were fully analyzed by two researchers (MTS, MFRST). The quality was evaluated on the basis of eight criteria, each yielding a score of zero or one.^5^ In this methodological scoring system for rating the studies, use of the following criteria received scores of one: (i) random sample or whole population; (ii) unbiased sampling frame (i.e. census data); (iii) adequate sample size (> 300 subjects); (iv) standard measurements; (v) outcomes measured by unbiased assessors; (vi) adequate response rate (> 70%) and subjects described as refusing treatment; (vii) confidence intervals and subgroup analysis; and (viii) study subjects described. The total score achieved could vary from zero (poor quality) to eight (high quality). Two researchers (SDF, MTS) recorded the contents of each selected article in a structured file that included: (i) names of the authors, year of publication and location of study; (ii) sample type; (iii) population characteristics; (iv) size of population; (v) screening tools; (vi) evaluators' background; (vii) occurrence of sample losses; (viii) prevalence rates of dementia and corresponding confidence intervals (95%); and (ix) sociodemographic characteristics of the population. We performed meta-analysis to pool the individual studies estimates and meta-regression to assess heterogeneity, both with random effects models, using the Meta-Analyst software.^6^

## RESULTS

**Figure 1.**
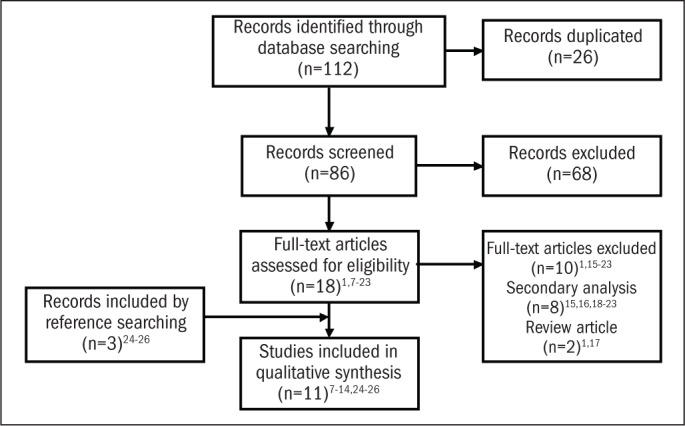
Fluxogram showing the process of article selection

**Figure 2.**
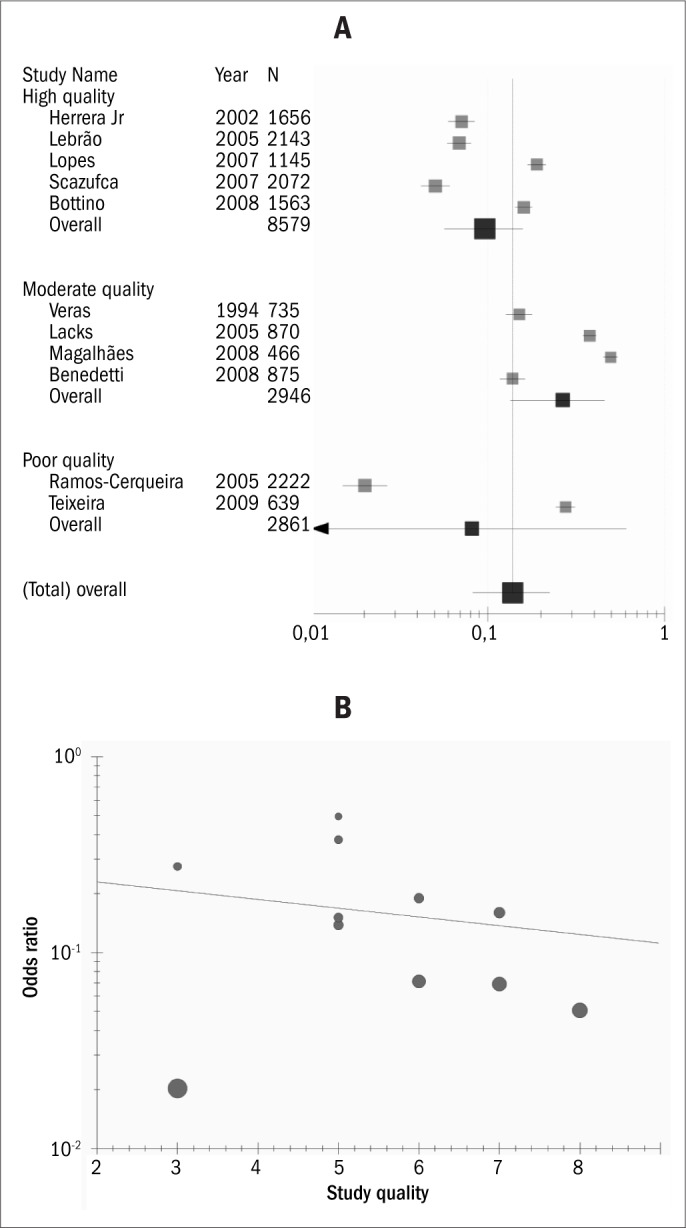
Meta-analysis (A) and meta-regression (B) on studies included

A total of 112 articles were identified through the online search, and eighteen^1,7-23^ were selected and fully analyzed (**Figure 1**). Only eight articles^7-14^ complied with the inclusion and exclusion criteria. Three additional papers^24-26^ were identified from the references. Five articles^7,10,11,18,26^ attained high-quality scores, according to the criteria adopted (≥ 6 points). The dementia rates reported in these five high-quality papers ranged from 5.1 to 19.0% (**Table 2**). A visual inspection of the meta-analysis suggested that there was high heterogeneity between the study results (**Figure 2A**). The meta-regression indicated that the prevalence was overestimated in the low-quality studies (**Figure 2B**). Because of this heterogeneity, we did not take the pooled data any further. The prevalence of dementia increased with age and was inversely related to the socioeconomic status and number of years of education (**Table 3**). Prevalence rates were higher among women.

**Table 2.** Details of studies selected for the systematic review on the prevalence of dementia among elderly populations in Brazil

First author and year published	Location	Subjects	Urban/rural	Screening and/or criterion tools	Prevalence (95% CI)	Quality score
**High-quality studies**						
Herrera Jr.,^23^	Catanduva (SP)	1,656	U	MMSE; PFAQ; DSM-IV	7.1 (6.0 - 8.5)	6
Lebrão e Laurenti^26,^	São Paulo (SP)	2,143	U	MMSE; PFAQ; GDS	6.9 (5.9 - 8.1)	7
Lopes et al.,^11^*	Ribeirão Preto (SP)	1,145	U	MMSE; FOME; IQCODE; B-ADL	19.0 (16.8 - 21.3)	6
Scazufca et al.,^10^	São Paulo (SP)	2,072	U	CSI-D; CERAD; GMS; HAS-DDS; DSM-IV	5.1 (4.2 - 6.1)	8
Bottino et al.,^7^	São Paulo (SP)	1,563	U	MMSE; FOME; IQCODE; B-ADL	16.0 (14.3 - 17.9)	7
**Moderate-quality studies**						
Veras e Murphy^25^*	Rio de Janeiro (RJ)	735	U	BOAS	15.1 (12.7 - 17.9)	5
Lacks et al.,^12^*	Santo Antônio de Pádua (RJ)	870	U	MMSE; PFAQ	37.7 (34.5 - 41.0)	5
Magalhães et al.,^8^	Santo Estevão (BA)	466	R	CAMDEX; CAMCOG	49.6 (45.0 - 54.1)	5
Benedetti et al.,^9^	Florianópolis (SC)	875	U	BOAS; IPAQ	13.8 (11.7 - 16.3)	5
**Poor-quality studies**						
Ramos-Cerqueira et al.,^24^	Pirajú (SP)	2,222	U	DSM-IV	2.0 (1.5 - 2.7)	3
Teixeira et al.,^14^	Caeté (MG)	639	U/R	MMSE; PFAQ; GDS; MINI; DSM-IV	27.5 (24.2 - 31.1)	3

* Dementia reported as cognitive impairment;

CI = confidence interval; U = urban; R = rural; MMSE = Mini Mental State Examination; FOME = Fuld Object Memory Evaluation; IQCODE = Informant Questionnaire on Cognitive Decline in the Elderly; B-ADL = Bayer-Activities of Daily Living scale; CAMDEX = Cambridge Examination for Mental Disorders; CAMCOG = cognitive section of CAMDEX; BOAS = Brazil Old Age Schedule; IPAQ = International Physical Activity Questionnaire; CSI-D = Community Screening Instrument for Dementia; CERAD = Consortium to Establish a Registry for Alzheimer's Disease; GMS = Geriatric Mental State; HAS-DDS = History and Aetiology Schedule Dementia Diagnosis and Subtype; DSM-IV = Diagnostic and Statistical Manual of Mental Disorders, 4th edition; PFAQ = Pfeffer Functional Activities Questionnaire; GDS = Geriatric Depression Scale; MINI = Mini International Neuropsychiatric Interview.

**Table 3.** Percentage of dementia among elderly populations in Brazil, distributed according to sociodemographic characteristics of the sample populations

Characteristics evaluated	Reference										
	High-quality studies					Moderate-quality studies				Poor-quality studies	
	Herrera Jr. et al.,^13,23^	Lebrão et al.,^4,27^	Lopes et al.,^11^	Scazufca et al.,^10^	Bottino et al.,^7^	Veras e Murphy^25^	Lacks et al.,^12^	Magalhães et al.,^8^	Benedetti et al.,^9^	Ramos-Cerqueira et al., ^24^	Teixeira et al., ^14^
Age (years)											
60-64	NR	3.6	10.6	NR	2.5	1.1	NR	44.0	NR	NR	NR
65-69	1.6	3.3	12.4	2.2	4.1					0.1	
70-74	3.2	6.5	17.1	2.0	7.1	1.4		52.7		1.2	
75-79	7.9	12.8	32.9	7.8	9.5					2.6	
80-84	15.1	19.5	40.7	13.6	13.3	3.2		53.4		3.1	
85-89	38.9	31.6	64.7	21.4	15.3					12.0	
≥90			66.7		42.3			86.7			
Gender											
Female	9.4	7.5	25.2	5.4	7.3	1.7	43.6	50.2	17.8	2.2	NR
Male	5.2	6.0	18.3	4.5	7.1	1.2	26.3	48.8	9.8	1.7	
Education (years)											
≥12	4.5	1.2	10.0	NR	3.1	NR	20.0	50.9	NR	NR	NR
9-11					4.1		13.0				
5-8		5.5	21.1		2.7		21.7				
1-4			26.6		6.7						
0	12.2	16.8	42.5		18.7		62.8	45.8			
Socioeconomic status											
A	5.4	NR	15.0	NR	2.9	NR	NR	NR	NR	NR	NR
B	7.2		14.6		5.2						
C	7.0		22.8		5.1						
D	6.7		32.1		10.7						
E	10.9		41.3		15.8						

NR = not reported.

## DISCUSSION

The present review presents an attempt to describe the prevalence of dementia among the elderly population in Brazil, derived from high-quality research papers.

Dementia rates have been determined in non-representative sample populations in which subjects were chosen according to opportunity and recruited from meeting places such as charity organizations and geriatric clinics. Studies based on such convenience populations were not taken into consideration in the present review, since the rates found have shown wide variability caused by selection bias. The rates in one systematic review^27^ ranged from 0.7 to 70.2%. For this reason, the present review only included population-based studies. A further limitation of many published studies relates to the size of the sample population, which may include as few as 40 participants.^27^ One criterion used in the present review was that for a study to be included, it should have a minimum of 300 participants.

Despite the high quality of the articles selected for this review, there were methodological disparities between them. For instance, the articles differed with regard to the age range of the population selected for the study. The age distribution in the sample population could affect the results, since in locations with a large number of individuals > 80 years old, the prevalence of dementia would probably be higher. The non-uniform age distribution of the sample populations considered in the studies reviewed may explain the variation in the prevalence of dementia that was detected. Similar assumptions may be made in relation to gender-based and socioeconomic status-based distributions, i.e. the prevalence of dementia is probably higher in populations that encompass large numbers of poor elderly women.

All the high-quality studies analyzed had been conducted in the State of São Paulo, in southeastern Brazil, an area that occupies a unique position in terms of development and living standards. Therefore, the findings regarding the prevalence of dementia cannot be generalized to other Brazilian regions. All of these five studies involved application of two or more validated screening tools. Although the use of different tests may improve the precision of the results, the possibility of divergence between evaluators is increased and the results may be distorted.

The prevalence of dementia in developing countries is often estimated from statistics from different locations, because of the lack or imprecision of local data. In developed countries, the prevalence of dementia apparently doubles for every five-year increase in age, and it typically varies from 3% at 70 years of age to 20-30% at 85 years of age.^1^ The results reviewed here suggest that the prevalence of dementia in the State of São Paulo is roughly compatible with that of many developed countries.

Canada presents one of the lowest rates of dementia in the world, with a prevalence of 4.2% among individuals ≥ 65 years of age.^28^ This relatively low incidence of the disease may be explained by a number of factors, including the existence of a first-class health system, the high quality of life among the elderly population, encouragement towards intellectual activities and the superior quality of research. In one of the Brazilian high-quality studies, the prevalence of dementia was lower than in Canada, in contrast to the other studies. The reason for the discrepancy between these studies is unclear, but may indicate a lack of standardization in the methodology used.

## CONCLUSIONS

Dementia was most prevalent among poor, illiterate, female and very elderly individuals. The overall prevalence of dementia among elderly Brazilians could not be estimated because of the wide variations reported. The studies reviewed may reflect the situation of southeastern Brazil and not the reality of the whole country. Methods with greater consistency should be used in clinical and epidemiological studies in order to assess the real extent of the problem of dementia in Brazil.
